# Comparison of Efficacy Between Transabdominal and Transthoracic Surgical Approaches for Siewert Type II Adenocarcinoma of the Esophagogastric Junction: A Systematic Review and Meta-Analysis

**DOI:** 10.3389/fonc.2022.813242

**Published:** 2022-04-29

**Authors:** Zonglin Li, Huaiwu Jiang, Jin Chen, Yifan Jiang, Yi Liu, Linxia Xu

**Affiliations:** ^1^ Department of Gastrointestinal Surgery, The Affiliated Hospital of Southwest Medical University, Luzhou, China; ^2^ Department of Gastrointestinal Surgery, Sichuan Mianyang 404 Hospital, Mianyang, China

**Keywords:** Adenocarcinoma of the esophagogastric junction (AEG), Siewert type II, transabdominal (TA), transthoracic (TT), surgical outcomes, oncological outcomes

## Abstract

**Background:**

The optimal surgical approach, whether transabdominal (TA) or transthoracic (TT), for Siewert type II adenocarcinoma of the esophagogastric junction (AEG) remains controversial. This study compares the efficacy of TA and TT surgical approaches for Siewert type II AEG.

**Methods:**

Studies comparing the surgical and oncological outcomes of TA and TT surgical approaches for Siewert type II AEG up to June 2021 were systematically searched on the Web of Science, PubMed, Embase, and Cochrane Library. A pooled analysis was performed for the available data regarding the baseline features, surgical, and oncological outcomes. The RevMan 5.3 software was used to perform the statistical analysis. Quality evaluation and publication bias were also conducted.

**Results:**

Twelve studies with a total of 2,011 patients, including 985 patients in the TA group and 1,026 patients in the TT group, were included in this study. In the pooled analysis, the surgical outcomes, namely, operative time (MD = −54.61, 95% CI = −123.76 to 14.54, P = 0.12), intraoperative blood loss (MD = −28.85, 95% CI = −71.15 to 13.46, P = 0.18), the number of dissected lymph nodes (MD = 1.90, 95% CI = −1.32 to 5.12, P = 0.25), postoperative complications (OR = 0.84, 95% CI = 0.65 to 1.07, p = 0.16), anastomotic leakage rate (OR = 1.02, 95% CI = 0.63 to 1.65, p = 0.93), and postoperative death rate (OR = 0.89, 95% CI = 0.46 to 1.72, p = 0.73), and the oncological outcomes, namely, overall recurrence rate (OR = 0.75, 95% CI = 0.37 to 1.50, p = 0.41), 3-year overall survival (OS) rate (OR = 1.19, 95% CI = 0.54 to 2.65, p = 0.66), and 5-year OS rate (OR = 1.21, 95% CI = 0.84 to 1.74, p = 0.30) of the two groups were all comparable.

**Conclusions:**

Both TA and TT surgical approaches are appropriate for Siewert type II AEG, and neither has a significant advantage in terms of short- and long-term outcomes. However, more high-quality randomized controlled trials are needed to confirm this conclusion.

## Introduction

The incidence of adenocarcinoma of the esophagogastric junction (AEG) has been greatly increasing in both western and eastern countries recently (1, 2). Based on Siewert’s classification, AEG were classified into three types: tumors with an epicenter of 1 to 5 cm above the esophagogastric junction (EGJ) are considered type I tumors; 1 cm above and 2 cm below the EGJ are considered type II tumors; and 2 to 5 cm below the EGJ is considered type III tumors (3). A consensus has been reached by most researchers that the surgical treatment of Siewert types I and III AEG should adhere to the principles of esophageal and gastric cancer, respectively (4). For the Siewert type I AEG, the transthoracic (TT) surgical approach performing subtotal esophagectomy with proximal gastrectomy (PG) is the standard surgical procedure (5). Conversely, Siewert type III AEG is treated as a proximal gastric cancer by performing total gastrectomy (TG) using a transabdominal (TA) surgical approach (6). However, the optimal surgical approach, whether TA or TT, for Siewert type II AEG, which is also called true carcinoma of the cardia, remains controversial. Usually, thoracic surgeons treat Siewert type II AEG according to the guidelines for esophagus cancer and consider the TT approach the preferred method. They point out that the TT approach has the merits of complete resection of the upper bound of the tumor, sufficient dissection of mediastinal lymph nodes (LNs), and little difficulty in anastomosis (7–9). However, gastrointestinal surgeons consider the TA approach the better choice, and they argue that the TA approach has the advantages of complete dissection of abdominal LNs, mild surgical trauma, fewer postoperative complications, and is especially suitable for elderly patients who have poor cardiorespiratory function (7, 10, 11).

In light of the above considerations, no consensus has been reached regarding the surgical approach for Siewert type II AEG. Therefore, further research is needed to assess the efficacy of TA and TT surgical approaches for Siewert type II AEG, but no large-scale randomized controlled trial (RCT) on this issue, especially aimed at Siewert type II AEG, is available to date. This meta-analysis evaluates the efficacy of TA and TT surgical approaches for Siewert type II AEG on the basis of the current published studies.

## Methods

This meta-analysis was carried out in line with the preferred reporting items for systematic reviews and meta-analysis (PRISMA) statement.

### Search Strategy

Studies systematically searched for studies comparing the surgical and oncological outcomes of TA and TT surgical approaches for Siewert type II AEG and published in English up to June 2021 were systematically searched on the Web of Science, PubMed, Embase, and Cochrane Library. The keywords used for the search were “adenocarcinoma”, “esophagogastric junction,” and “surgery.” Thus, the following search string was used across the above databases: “‘adenocarcinoma’ OR ‘tumor’ OR ‘cancer’” AND “‘esophagogastric junction’ OR ‘cardia’” AND “‘surgery’ OR ‘surgical approach’ OR ‘treatment’ OR ‘transabdominal’ OR ‘transhiatal’ OR ‘transthoracic’”. Articles from previously published reviews and meta-analyses were also checked for potential articles. The search was conducted independently by two authors (ZL and HJ) and was last performed on June 19, 2021.

### Study Selection and Data Extraction

The included studies met the following criteria: (1) comparative studies on the surgical and oncological outcomes of TA and TT surgical approaches for Siewert type II AEG, and (2) the original research published in English. The exclusion criteria were as follows: (1) studies published as reviews, case reports, letters, animal studies, meeting abstracts and protocols of RCT; (2) not comparative studies between TA and TT surgical approaches for Siewert type II AEG; and (3) articles with a mixed study population, resulting in inaccessible analysis for Siewert type II AEG patients.

Two reviewers (ZL and HJ) carried out the screening and extraction processes independently. First, studies were screened by titles and abstracts. Then, the full texts of the potential studies were checked. For eligible articles, the following information from each article was recorded: the first author, publication year, country, study design, study interval, study object, and sample size. Furthermore, the following clinicopathological parameters were extracted from these studies: sex, age, body mass index (BMI), American Society of Anesthesiologists (ASA) score, pathological stage, histologic type, neoadjuvant chemoradiotherapy, postoperative chemoradiotherapy, operation time, intraoperative blood loss, the number of retrieved LNs, postoperative complications, anastomotic leakage rate, postoperative death rate, overall recurrence rate, 3-year overall survival (OS) rates, and the 5-year OS rate. Results were checked by a third author (LX).

### Risk of Bias Assessment

The quality of the selected studies was assessed in accordance with the Cochrane Handbook. Biases, namely, selection, performance, detection, attrition, reporting, and others, were evaluated. Outcomes were summarized using a bias graph.

### Statistical Analysis

The odds ratio (OR) and mean difference (MD) with a 95% confidence interval (CI) were used to evaluate the dichotomous and continuous variables, respectively. For studies that only reported median and range, data were converted into mean and standard deviation (12). Heterogeneity among studies was assessed using *χ* ^2^ and *I* ^2^ statistics. *I* ^2^ <50% indicated acceptability heterogeneity, and the fixed-effects model was used. Otherwise, the random-effects model was performed. Funnel plots were conducted to assess publication bias. A p-value of <0.05 was considered significant. All statistical analyses were performed using the RevMan 5.3 software (Cochrane, London, UK).

## Results

### Characteristics of Studies

**Figure 1:**
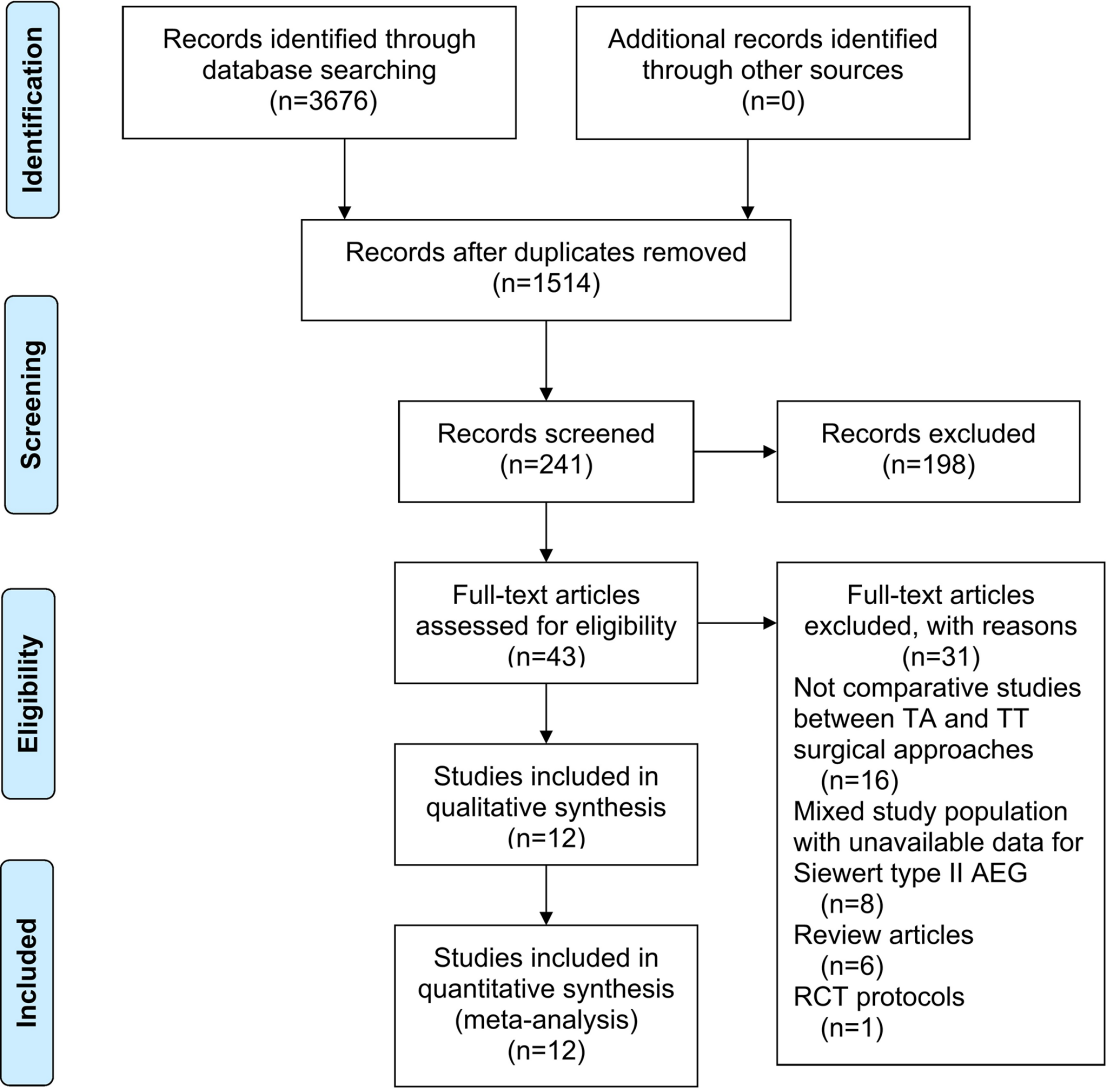
PRISMA flowchart of literature search and selection process. PRISMA, preferred reporting items for systematic review and meta-analysis; TA, transabdominal; TT, transthoracic; AEG, adenocarcinoma of esophagogastric junction; RCT, randomized controlled trial.

**Figure 2:**
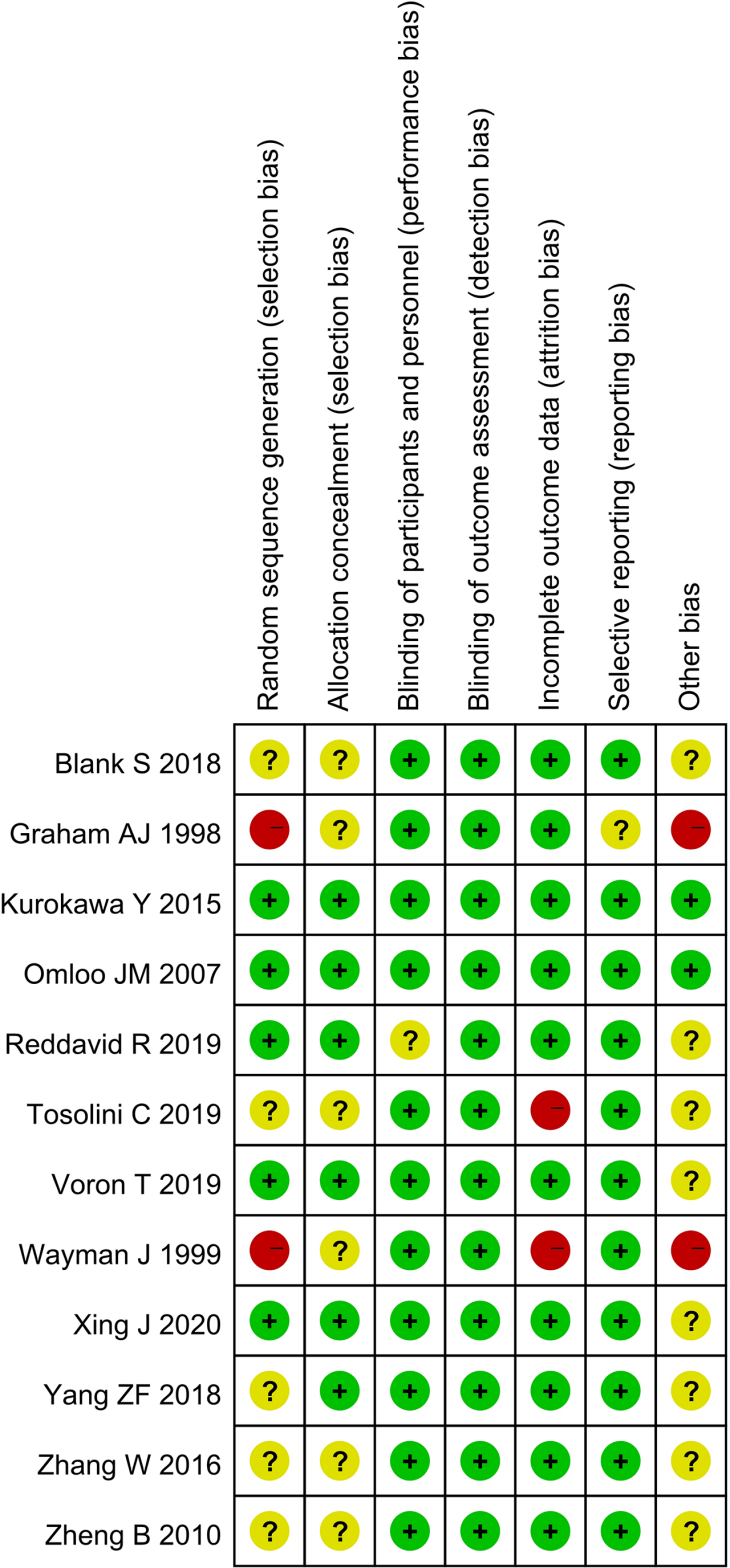
Risk of bias summary for the included studies.

A total of 3,676 studies were identified. Twelve studies (8, 10, 13–22), namely, 10 retrospective studies and 2 RCTs, were ultimately included in this meta-analysis. Five multicenter studies were obtained. The details of the selection procedures conformed to the PRISMA flowchart (**Figure 1**). General information from the included studies is summarized in **Table 1**. The total number of included patients with Siewert type II AEG was 2,011 (985 in the TA group and 1,026 in the TT group). These studies were from 8 countries (i.e., Canada, UK, Netherlands, China, Japan, Germany, Italy, and France) and were published from 1998 to 2020. The sample size ranged from 40 patients to 331 patients. According to the Cochrane Handbook, twelve studies were at a slight or moderate risk of bias. The items evaluated for each study are shown in **Figure 2**.

**Table 1:** Characteristics of included studies.

Reference	Published year	Country	Study interval	Study design	Study object	Sample size (TA : TT)	Outcomes
Graham et al. (13)	1998	Canada	1985-1997	M;R	NA	119:32	5,6,9
Wayman et al. (14)	1999	UK	1991-1995	S;R	NA	20:20	1,2,3,4
Omloo et al. (15)	2007	Netherlands	1994-2000	M;RCT	NA	52:63	9
Zheng et al. (16)	2010	China	1994-2003	S;R	NA	47:284	1,2,3,4,5,6,8,9
Kurokawa et al. (17)	2015	Japan	1995-2003	M;RCT	pT1-4N0-3	52:43	9
Zhang et al. (18)	2016	China	2006-2009	S;R	pT1-4N0-3	101:69	9
Blank et al. (8)	2018	Germany	2001-2015	S;R	NA	186:56	3,4,5,6,7,8,9
Yang et al. (19)	2018	China	2004-2014	S;R	pT1-4N0-3	77:81	1,2,3,4,5,6,9
Reddavid et al. (20)	2019	Italy	2000-2017	M;R	NA	60:140	3,4,5,6,9
Tosolini et al. (21)	2019	Germany	2000-2013	S;R	NA	179:91	3,4,5,6,7
Voron et al. (22)	2019	France	1997-2010	M;R	pT1-4N0-3	64:119	3,4,6,7,8,9
Xing et al. (10)	2020	China	2009-2018	S;R;PSM	pT3-4N0-3	28:28	1,2,3,4,5,7,8

TA, transabdominal; TT, transthoracic; S, single center; M, multicenter; R, retrospective study; PSM, propensity score matching; RCT, randomized controlled trial; NA, not available; 1, operative time; 2, intraoperative blood loss; 3, the number of dissected lymph nodes; 4, postoperative complications; 5, anastomotic leakage; 6, postoperative death; 7, overall recurrence rate; 8, 3-year overall survival; 9, 5-year overall survival.

### Patient- and Tumor-Related Baseline Characteristics

**Figure 3:**
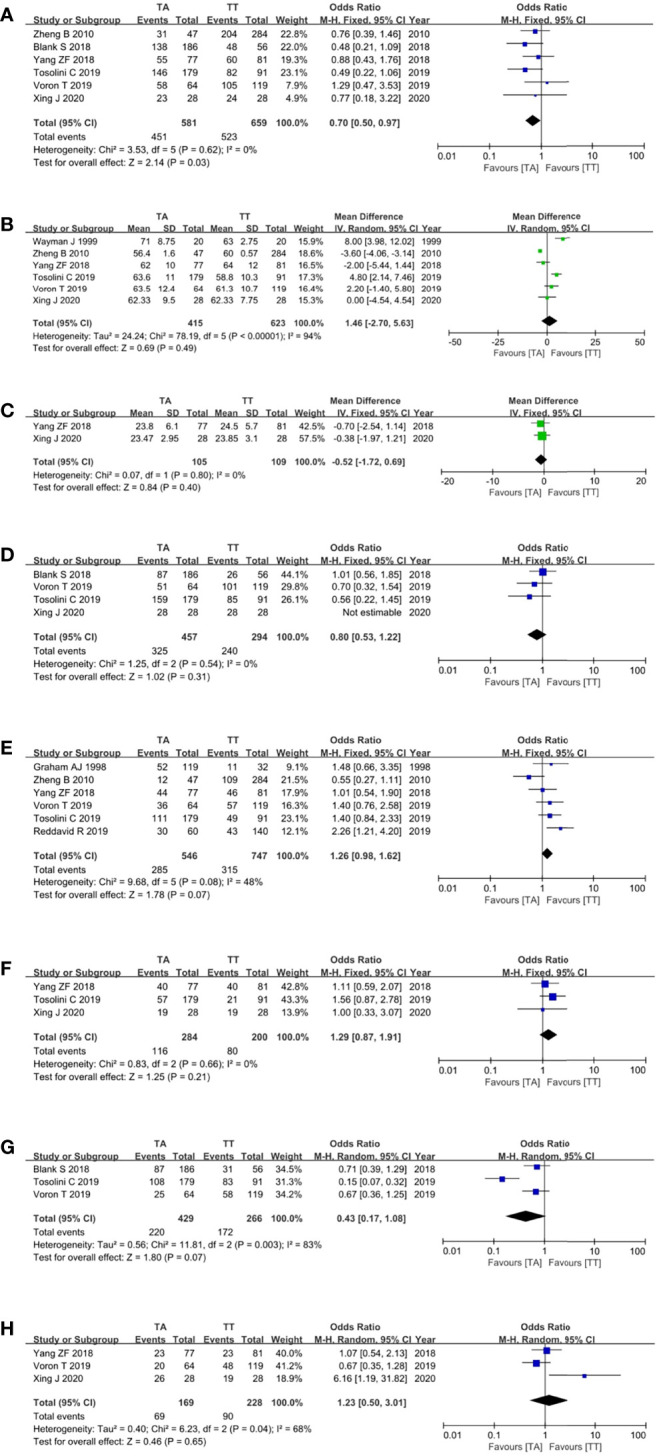
Forest plots showing the assessment of baseline features, namely, **(A)** sex, **(B)** age, **(C)** body mass index, **(D)** American Society of Anaesthesiologists score, **(E)** pathological stage, **(F)** histologic type, **(G)** neoadjuvant chemoradiotherapy, and **(H)** postoperative chemoradiotherapy. TA, transabdominal, TT, transthoracic.

For the patient- and tumor-related variables, sex (male and female), age (mean ± SD), BMI (mean ± SD), ASA score (ASA 1/2 and ASA 3/4), pathological stage (stages 1/2 and 3/4), histologic type (differentiated and other types), neoadjuvant chemoradiotherapy (with and without), and postoperative chemoradiotherapy (with and without) were analyzed. As shown in **Figure 3**, in addition to gender (p = 0.03), all baseline parameters in the TA and TT groups were not statistically significant (p >0.05).

### Surgical Outcomes

**Figure 4:**
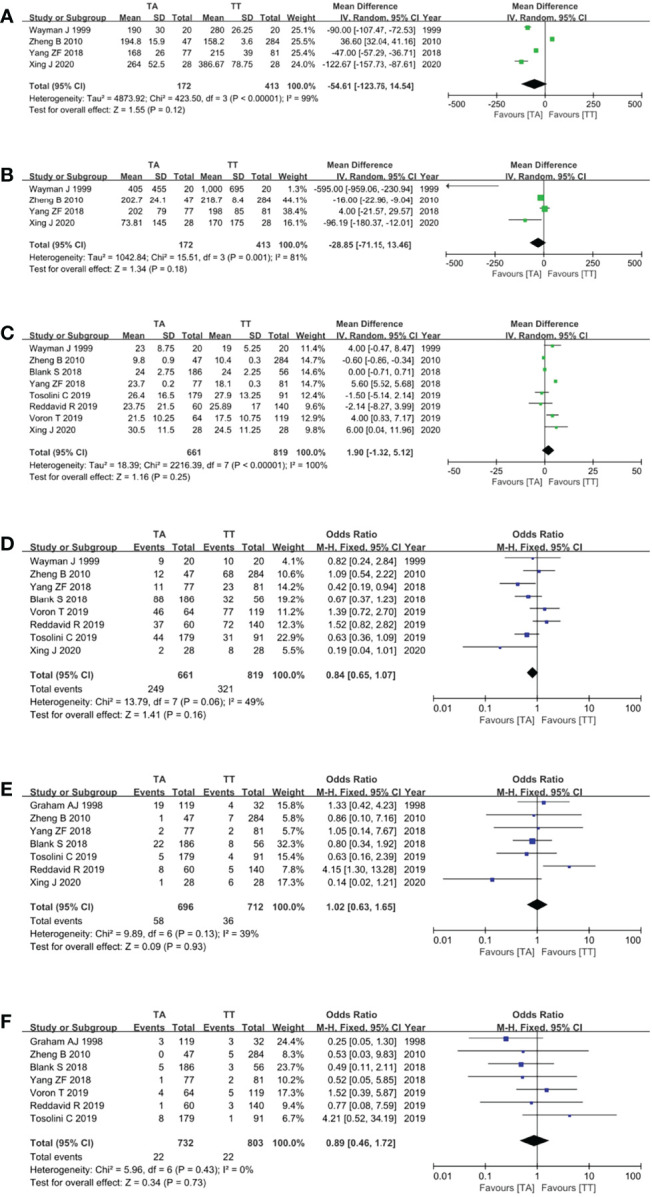
Forest plots showing the assessment of surgical outcomes, namely, **(A)** operative time, **(B)** intraoperative blood loss, **(C)** the number of dissected lymph nodes, **(D)** postoperative complications, **(E)** anastomotic leakage rate, and **(F)** postoperative death rate. TA, transabdominal; TT, transthoracic.

Four studies (10, 14, 16, 19) reported the operation time and showed that the TA approach took a shorter time, but the pooled analysis showed no difference between the TA and TT groups (MD = −54.61, 95% CI = −123.76 to 14.54, P = 0.12) (**Figure 4A**). Four studies (10, 14, 16, 19) reported intraoperative blood loss and there was a trend that the TA approach was related to less intraoperative blood loss with no statistical difference (MD = −28.85, 95% CI = −71.15 to 13.46, P = 0.18) (**Figure 4B**). Eight studies (8, 10, 14, 16, 19–22) reported the number of retrieved LNs (MD = 1.90, 95% CI = −1.32 to 5.12, P = 0.25) (**Figure 4C**), eight studies (8, 10, 14, 16, 19–22) reported the postoperative complications (OR = 0.84, 95% CI = 0.65 to 1.07, p = 0.16) (**Figure 4D**), seven studies (8, 10, 13, 16, 19–21) reported the anastomotic leakage rate (OR = 1.02, 95% CI = 0.63 to 1.65, p = 0.93) (**Figure 4E**), seven studies (8, 13, 16, 19–22) reported the postoperative death rate (OR = 0.89, 95% CI = 0.46 to 1.72, p = 0.73) (**Figure 4F**), and there were no differences between the two groups for these surgical outcomes (p >0.05).

### Oncological Outcomes

**Figure 5:**
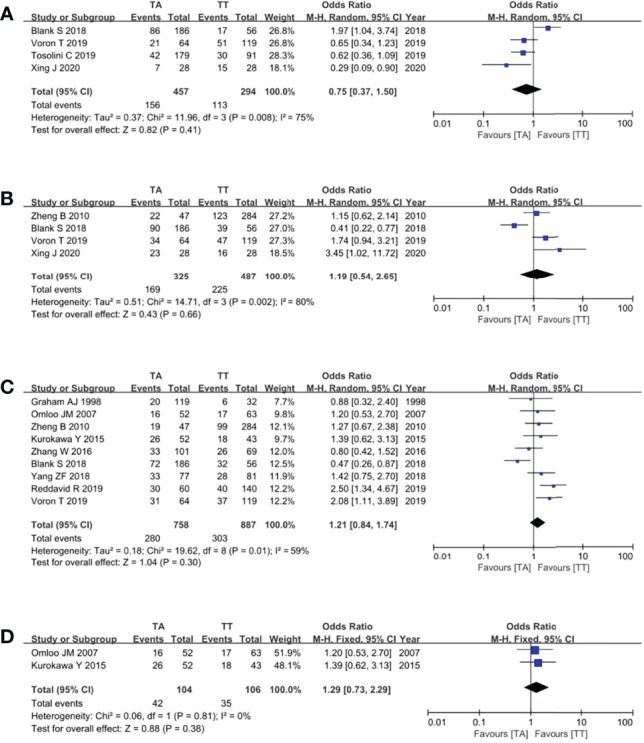
Forest plots showing the assessment of oncological outcomes, namely, **(A)** overall recurrence rate, **(B)** 3-year OS rate, **(C)** 5-year OS rate, and **(D)** 5-year OS rate based on RCTs. TA, transabdominal; TT, transthoracic; OS, overall survival; RCT, randomized controlled trial.

Four studies (8, 10, 21, 22) reported the overall recurrence rate. These studies expatriated and compared the recurrence rate and type between the two groups. Recurrence patterns were classified as a recurrence of the primary site, mediastinum, peritoneum, LNs, liver, lung, bone, and combined metastasis. The pooled analysis showed no significant difference in the overall recurrence rates of the TA and TT groups (OR = 0.75, 95% CI = 0.37 to 1.50, p = 0.41) (**Figure 5A**).

The primary outcome of this study was the assessment of the OS rate of TA and TT surgical approaches for Siewert type II AEG patients. Ultimately, four studies (8, 10, 16, 22) reported the 3-year OS rates, and the pooled analysis showed no significant difference in the 3-year OS rate between the two groups (OR = 1.19, 95% CI = 0.54 to 2.65, p = 0.66) (**Figure 5B**). Nine studies (8, 13, 15–20, 22) reported the 5-year OS rates, and the meta-analysis of pooled analysis showed that the 5-year OS rates of the two groups were similar (OR = 1.21, 95% CI = 0.84 to 1.74, p = 0.30) (**Figure 5C**). Two RCTs (15, 17) reported the 5-year OS rates, and the pooled analysis still showed no difference between the two groups (OR = 1.29, 95% CI = 0.73 to 2.29, p = 0.38) (**Figure 5D**).

However, there may be a trend that the TA approach was related to better oncological outcomes, with an overall recurrence rate of 34.1% (156/457) vs 38.4% (113/294), 3-year OS rates of 52.0% (169/325) vs 46.2% (225/487), and 5-year OS rates of 36.9% (280/758) vs 34.2% (303/887) for TA and TT groups, respectively.

### Publication Bias

**Figure 6:**
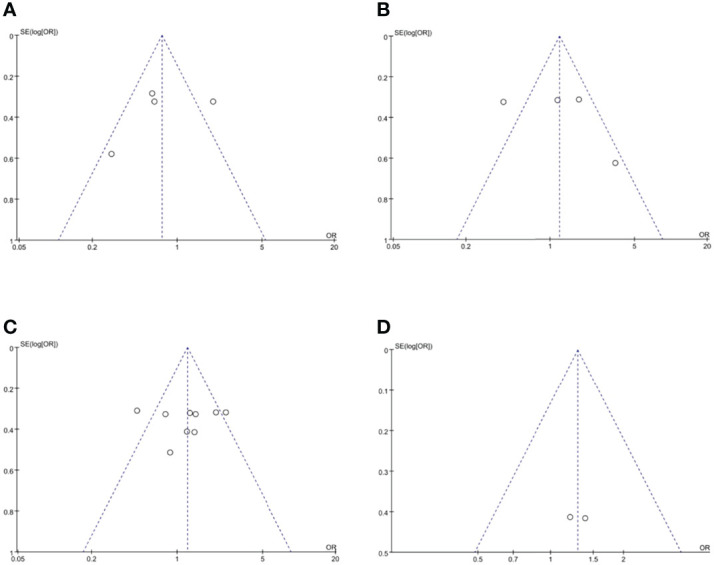
Funnel plots of publication bias, namely, **(A)** overall recurrence rate, **(B)** 3-year OS rate, **(C)** 5-year OS rate, and **(D)** 5-year OS rate based on RCTs. RCT, randomized controlled trial.

Funnel plots were used to assess the potential publication bias in the meta-analysis. As shown in **Figure 6**, these funnel plots were symmetrical, which showed a low risk of publication bias in this study.

## Discussion

AEGs are biologically aggressive and most are diagnosed at an advanced stage, resulting in poor prognosis. Currently, surgery remains the mainstay treatment for resectable AEG. According to the 8th edition of the American Joint Committee on Cancer (AJCC) staging, tumors involving GEJ with an epicenter of ≤2 cm in the proximal stomach (Siewert types I and II) are considered esophageal cancer, and tumors with an epicenter located >2 cm in the proximal stomach (Siewert type III) are staged as gastric carcinoma (4). Currently, a worldwide consensus exists that subtotal esophagectomy with PG by TT surgical approach and TG by TA surgical approach are the standard surgical procedures for Siewert type I and III AEG, respectively (4–6). However, due to its unique anatomic location, the optimal surgical approach, whether TA or TT, for Siewert type II AEG remains controversial.

The TA and TT approaches are two major surgical approaches for treating Siewert type II AEG, and the two approaches both have merits and weaknesses. The TT approach has the advantages of wide surgical vision, complete resection of the upper bound of the tumors, effective dissection of mediastinal LNs, and little difficulty in anastomosis, while the weaknesses include severe surgical trauma, incomplete dissection of abdominal LNs, and influence on the respiratory and circulatory systems (7–9). The TA approach has the merits of mild surgical trauma, complete dissection of abdominal LNs and low requirements on the physical condition of patients, while the weaknesses include insufficient resection of mediastinal LNs, limitation on sufficient proximal surgical margin, and difficulty in anastomosis (7, 10, 11). According to the pooled analysis in our study, the operation time, intraoperative blood loss, number of retrieved LNs, postoperative complications, anastomotic leakage rates, and postoperative death rates were all comparable between the TA and TT groups (p >0.05). These results suggest that the surgical outcomes of the two approaches are similar and that one offers no significant advantage over the other in terms of short-term outcomes.

Sufficient lymphadenectomy is required for AEG patient survival, and LN status is a stronger prognostic factor for AEG patient survival than any other factor. However, the lymphatic pathways may advance both up into the mediastinum and down into the abdomen for Siewert type II AEG, and therefore, it is difficult for thoracic surgeons to resect the abdominal LNs and gastrointestinal surgeons to resect the mediastinal LNs. Many retrospective studies have recommended lymphadenectomy of mediastinal LNs for patients with Siewert type II AEG (23, 24). However, a recent study reported that the dissection of abdominal LNs rather than mediastinal LNs was an important prognostic factor for Siewert type II AEG (25). Based on our meta-analysis, there is no significant difference in the number of dissected LNs between the TA and TT groups (p = 0.25), so it is that the possible the two approaches are both acceptable in terms of lymphadenectomy. Noteworthily, several studies have reported that most metastatic LNs were the paracardial and lesser curvature LNs (particularly in Nos.1, 2, 3 and 7 LN stations) (26, 27) and patients with metastasis of mediastinal LNs had a poor prognosis even if the complete dissection of mediastinal LNs was done (28).

Oncological outcomes are the primary outcomes of this study. According to the pooled analysis, although there is a slight trend that the TA approach was related to better oncological outcomes, the overall recurrence rate, 3-year OS rates, and 5-year OS rates are comparable between the TA and TT groups (p >0.05). In 2020, another meta-analysis pointed out that the TA approach may be more appropriate for Siewert type II AEG because the TA approach was related to less intraoperative blood loss, shorter hospital stays, and longer 3- and 5-year OS rates (29). Nevertheless, this meta-analysis includes 3 Chinese articles out of 11 included articles and missed 3 English articles (13, 14, 18) by the date the authors performed the last search in the databases, so its conclusion may not credible be enough. Recently, a high-quality study comparing the efficacy of TA and TT surgical approaches for Siewert type II/III AEG following neo-adjuvant chemotherapy also reported that there is no difference in the short- and long-term outcomes between TA and TT approaches (30). However, no large-scale RCT on this issue, especially aimed at Siewert type II AEG, is available to date. Expectantly, a multicenter RCT (DRKS00016923) (31) performed by multiple countries (i.e., Germany, Switzerland, Netherlands, Sweden, Ireland, and France) is ongoing, and the results of this study may give a clear conclusion on this issue in the near future.

### Conclusions

Despite the limitations of the included studies, this meta-analysis concludes that both the TA and TT surgical approaches are acceptable for Siewert type II AEG, with no significant difference in short- and long-term outcomes. However, more high-quality randomized controlled trials are needed to confirm this conclusion.

## Data Availability Statement

The original contributions presented in the study are included in the article/supplementary material. Further inquiries can be directed to the corresponding author.

## Author Contributions

ZL and LX made substantial contributions to conception and design for this work. ZL, HJ, and LX collected all the data. ZL and LX were the major contributors in writing the manuscript. HJ, JC, YJ, and YL performed critical revision for important intellectual content. All authors listed have made a substantial, direct, and intellectual contribution to the work and approved it for publication.

## Funding

This work was supported by the Scientific Research Project of Southwest Medical University (No. 2020ZRQNB026).

## Conflict of Interest

The authors declare that the research was conducted in the absence of any commercial or financial relationships that could be construed as a potential conflict of interest.

## Publisher’s Note

All claims expressed in this article are solely those of the authors and do not necessarily represent those of their affiliated organizations, or those of the publisher, the editors and the reviewers. Any product that may be evaluated in this article, or claim that may be made by its manufacturer, is not guaranteed or endorsed by the publisher.
